# Current Trends of Nanobiosensors for Point-of-Care Diagnostics

**DOI:** 10.1155/2019/2179718

**Published:** 2019-10-23

**Authors:** Naumih M. Noah, Peter M. Ndangili

**Affiliations:** ^1^School of Pharmacy and Health Sciences, United States International University-Africa, P.O. Box 14634-00800, Nairobi, Kenya; ^2^Department of Chemical Science and Technology (DCST), Technical University of Kenya, P.O. Box 52428-00200, Nairobi, Kenya

## Abstract

In order to provide better-quality health care, it is very important that high standards of health care management are achieved by making timely decisions based on rapid diagnostics, smart data analysis, and informatics analysis. Point-of-care testing ensures fast detection of analytes near to the patients facilitating a better disease diagnosis, monitoring, and management. It also enables quick medical decisions since the diseases can be diagnosed at an early stage which leads to improved health outcomes for the patients enabling them to start early treatment. In the recent past, various potential point-of-care devices have been developed and they are paving the way to next-generation point-of-care testing. Biosensors are very critical components of point-of-care devices since they are directly responsible for the bioanalytical performance of an essay. As such, they have been explored for their prospective point-of-care applications necessary for personalized health care management since they usually estimate the levels of biological markers or any chemical reaction by producing signals mainly associated with the concentration of an analyte and hence can detect disease causing markers such as body fluids. Their high selectivity and sensitivity have allowed for early diagnosis and management of targeted diseases; hence, facilitating timely therapy decisions and combination with nanotechnology can improve assessment of the disease onset and its progression and help to plan for treatment of many diseases. In this review, we explore how nanotechnology has been utilized in the development of nanosensors and the current trends of these nanosensors for point-of-care diagnosis of various diseases.

## 1. Introduction

**Figure 1:**
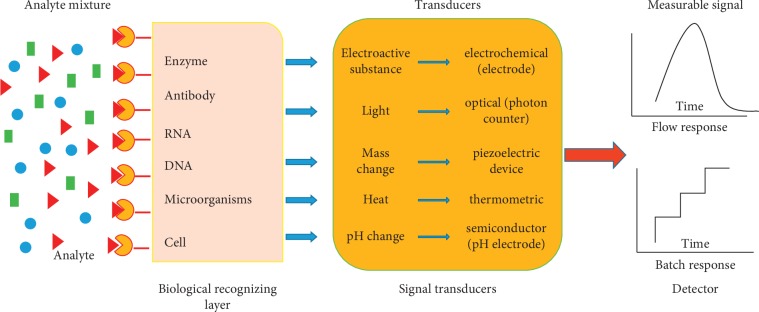
A schematic diagram showing a typical biosensor with all its components.

Better-quality health management is crucial in providing better health care [1–3], and higher standards of health care management can be achieved by making timely decisions based on rapid diagnostics, smart data analysis, and informatics analysis [3]. This calls for smart therapeutics, analytical tools, and diagnostics systems in order to enhance the health wellness [3, 4]. Effective management of a disease progression and monitoring evaluation which is important for epidemic understanding and management of the disease depends on the optimization of therapeutics [3]. Thus, development of smart diagnostic systems for personalized health care such as point-of-care devices is imperative. Point-of-care testing ensures fast detection of analytes near to the patients facilitating a better disease diagnosis, monitoring, and management. It also enables quick medical decisions since the diseases can be diagnosed at an early stage which leads to improved health outcomes for the patients enabling them to start early treatment [5]. Numerous potential point-of-care devices have been developed in recent years which are paving the way to next-generation point-of-care testing [6]. Biosensors, which are analytical devices that convert or transduce a biological response into a quantifiable signal [7], are very critical component of point-of-care devices since they are directly responsible for the bioanalytical performance of an essay [6]. The quantifiable signal may be optical, electrochemical, piezoelectric, or thermal, as shown in Figure 1. Electrochemical biosensors have attracted a lot of attention in the recent past due to their high sensitivity, accuracy, low detection limits, and great potential in real-sample analysis [8]. They have been explored for their prospective point-of-care applications necessary for personalized health care management [3, 9] since they usually estimate the levels of biological markers or any chemical reaction by producing signals mainly associated with the concentration of an analyte and hence can detect disease causing markers such as body fluids [7, 10]. Their high selectivity and sensitivity have allowed for early diagnosis and management of targeted diseases; hence, facilitating timely therapy decisions [3] and with combination of the biosensors with nanotechnology can improve assessment of the disease onset and its progression and help to plan for treatment of many diseases [7].

The field of nanotechnology which studies the manipulation of matter on atomic and molecular levels involves production and application of the physical, chemical, and biological systems at the 1–100 nanometer scale. These materials, usually known as nanoparticles or nanomaterials, are transforming the scientific world mainly because of their exceptional physical, chemical, and biological properties, in comparison to their bulk counterparts [11] and have found a wide range of applications especially in the field of biomedical, optical, medical imaging, catalysis, and electronics [12–15]. They are well suited for biosensing due to their improved catalytic properties, electron transfer, and their capability to be used in biomolecule labeling and adsorption [16]. The unique physicochemical properties of nanoparticles have led to the development of biosensors such as nanosensors for point-of-care disease diagnosis. Their small size usually improves performance of other methods such as electrochemical and enzymatic biosensors by increasing the electron transfer rates as well as by shortening enzyme-to-electrode distances [17]. Noble metal nanoparticles can also enhance localized surface plasmon resonance (SPR) and accordingly can improve optical biosensors [18]. For example, the color changes of these nanoparticles due to their interparticle plasmon coupling have been widely used in biosensors based on aggregation of the nanoparticles [19–24]. This review explores the recent trends of these nanosensors in point-of-care diagnostics.

## 2. Various Nanosensors for Point-of-Care Diagnostics

### 2.1. Nanosensors for Point-of-Care Diagnosis of Cancer

Cancer is one of the leading causes of death, not only in the developing countries but accounts for one in every seven deaths in the world [25, 26]. There are over 200 types of cancers, but the most common types include breast cancer, ovarian cancer, prostate cancer, esophageal cancer, colorectal cancer, lung cancer, bladder cancer, kidney cancer, lymphoma, skin cancer, liver cancer, pancreatic cancer, and thyroid cancer [27]. Breast cancer and ovarian cancer are the commonly reported life-threatening type of cancers in women. About 180,000 new cases of breast cancer are diagnosed every year, while 238,000 women are diagnosed with ovarian cancer worldwide, out of which 151,000 deaths occur [28]. Early screening and diagnosis are recognized practices for improving the likelihood of cancer survival and recovery, thereby leading to significant decrease in cancer mortality [29].

Cancer diagnosis usually involves detecting symptoms and characteristics that signify the presence of anomalies, which include biomarkers [30] such as nucleic acids, proteins, sugars, whole cells, cytokinetic parameters, cytogenetic parameters, and small metabolites found in body fluids [29]. Blood contains a wide variety of protein biomarkers with potential applications in early cancer diagnostics and detection [31]. However, conventional blood tests for early detection of cancer biomarkers yield low sensitivities owing to the biomarkers' low concentration in the cardiovascular system [32]. To effectively detect biomarkers in blood, sensors whose sensitivity allows them to detect biomarkers at a million times lower than the concentration of other blood proteins are required [31]. Currently, there exist limited devices for early screening, diagnosis, and monitoring cancer progress [33]. The few devices that already exist are costly, time-consuming, use sophisticated instrumentation, their use require centralized or hospital-based laboratories and high expertise in operating them [26]. New devices are needed for ultrasensitive and precise point-of-care diagnostics for early screening and detection of cancer biomarkers even at the bedside [33]. An ideal point-of-care diagnostic device is one that is portable and assures reliability. This calls for research on development of new devices that would offer continuous, cost-effective real-time in vivo monitoring of cancer, which would provide early diagnosis, drug efficacy, and effective drug delivery [34]. The use of nanotechnology for drug diagnosis, drug delivery, and cancer therapy has enabled the use of nanomaterials for extraction and detection of specific tumor biomarkers [35], circulating tumor cells, or extracellular vesicles shed by the tumor [36]. Recent research has seen emergence of nano- and microfabrication-based technologies that are integrated with different sensing platforms [33] and molecular communication [32]. Several authors have reported different sensor fabrications for cancer biomarkers. In this section, the recent advances in point-of-care nanosensors for cancer biomarkers are reviewed.

Mobile nanosensors for early detection of cancer in blood vessels were proposed and reported in 2018 [32]. The authors of this work focused on cancer cells located in particular regions of the blood vessel, and their detection was based on production and emission of biomarkers which signify an anomaly in the cardiovascular system. By targeting particular regions of the blood vessel, the authors ensured a close vicinity of the sensor to a high concentration of cancer cells and consequently cancer biomarkers. The authors were able to overcome the challenges met with conventional blood tests for early detection of cancer biomarkers owing to their low concentration in randomly picked blood samples.

**Figure 2:**
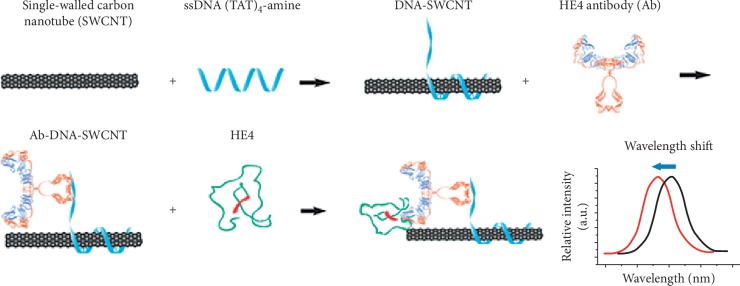
A schematic design of Ab-DNA-SWCNT complex synthesis and in vitro characterization of the proposed optical nanosensor for HE4. Reproduced from Williams et al. [38], an open-access article distributed under the terms of the Creative Commons Attribution-Noncommercial License which permits use, distribution, and reproduction in any medium, so long as the resultant use is not for commercial advantage and provided the original work is properly cited.

Mohanty and coworkers [37] reported the use of silicon nanochannel field effect transistor biosensor devices for breast cancer diagnosis and screening. The use of the silicon nanochannels allowed detection of single molecules due to high surface to volume ratios and assured high sensitivities due to their excellent electrical properties and small dimensions. In another research by Williams et al. [38], they developed a carbon nanotube-based implantable sensor for in vivo optical detection of human epididymis protein 4 (HE4), which is a biomarker for ovarian cancer. The sensor was based on near infrared emission properties of single-walled carbon nanotube (SWCNT) to transduce HE4—antibody binding activities. By modulating the SWCNT emission wavelength, the carbon nanotube-antibody complex was able to specifically detect HE4 and differentiated high-grade serious ovarian carcinoma from control patients, as demonstrated in Figure 2. The authors further modified the sensor into an implantable form and surgically inserted it into mice. The results obtained showed great success in quantitative detection of exogenously derived HE4 and endogenously detected HE4 in orthotopic murine models of ovarian cancer to differentiate HE4-producing models from biomarker-deficient models. Their results demonstrated that the device showed great potential in early detection of ovarian cancer biomarker localized in a region within the body. It can also monitor the progression of the cancer response to medication. However, the authors did not mention or investigate the effects of bioaccumulation of the carbon nanotubes in the human body. Moreover, the applicability of the fabricated sensor for onsite cancer diagnosis, cost, and accessibility to rural patients are aspects that require further research, improvements, and tested for potential use.

### 2.2. Nanosensors for Point-of-Care Diagnosis of Diabetes

Diabetes is a fast developing problem currently affecting millions of people worldwide [39]. It can lead to several serious complications such as lower limb amputations, blindness, cardiovascular diseases [39], and diabetic kidney disease [40]. Though diabetes has no cure, patients can reduce its complications by tightly monitoring and controlling the blood glucose levels [7, 39, 41]. Early detection and strategies to prevent the progression of diabetes would make a big difference for the patients and would also be economically beneficial for a resource-constrained country [40]. Studies have shown that the control of blood glucose level in the normal range, commonly found in the range of 4.9–6.9 mM in healthy individuals, can help amelioration of microvascular (nephropathy, neuropathy, and retinopathy) and macrovascular (coronary artery disease and stroke) complications [7]. In order to attain optimal control of the glucose levels, currently, the patients usually obtain a small blood sample, typically via a finger prick, which is then placed onto a sensor test strip and then read by a handheld electronic reader, which reports the blood glucose concentration [39, 41]. The glucose sensors which have been used since the 1970s are based on electrochemical enzymatic measurements with screen printed electrodes [41, 42] and provide rapid and accurate measurements of blood glucose without the need for laboratory analysis [39, 41, 42]. Other glucose biosensors reported in literature include fluorescent biosensors.

**Figure 3:**
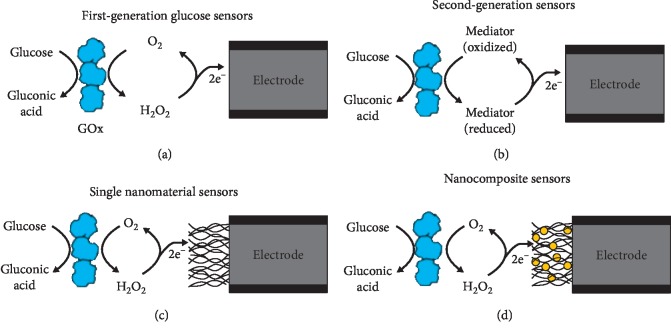
A schematic diagram showing the evolvement of glucose sensors from the first generation to the nanostructured materials used in glucose sensors. (a, b) Standard glucose oxidase- (GOx-) based electrochemical biosensors utilizing a GOx layer to recognize glucose and generate an electrochemical signal which is transferred from the enzyme through O_2_ reduction to H_2_O_2_. (c, d) shows the incorporation of nanomaterials such as CNTs or nanocomposites consisting of multiple nanomaterials into the sensors in order to increase surface area, improve catalytic action, modify operating parameters, and improve electron transfer from the enzyme to the electrode [39].

However, several limitations to the mentioned approaches have been reported. For example, the sampling process is painful and analysis cannot be done if patient is not awake and missing large fluctuations between sampling time points [39, 42–44]. Also, early diagnostic in remote and resource-constrained setting is challenging with no access to expensive well-equipped clinical laboratories and trained medical personnel [40]. Because of these limitations in diagnostic methods, substantial research efforts are focusing on developing enhanced methods to measure blood glucose levels. Development of cost-effective and easy to implement diagnostic tools remains an important goal in global health [40]. As a result, new commercial products for continuous glucose monitoring where discrete blood sampling time points during the course of the day is done providing the diabetics with acceptable data for the control of glucose in the blood [39]. Another promising approach is the detection of biomarkers from accessible body fluids with point-of-care biosensors since they can potentially improve patient care through real-time and remote health monitoring [40]. Nanotechnology research involving nanosensors and nanomaterials is also being directed towards continuous monitoring, and it has impacted these efforts by increasing the surface area of sensors, improving the catalytic properties of electrodes and providing nanoscale sensors [39], and is being used in these point-of-care devices for diagnosis of diabetes. For example, catalytic nanomaterials such as carbon nanotubes [45], graphene [46], electrospun nanofibers, and quantum dots have been incorporated in biosensors to enhance their sensitivity, response time, and limit of detection [47]. A wide range of new biosensors with nanomaterials such as lab-on-chip and nanosensor devices are currently being developed for in vivo and in vitro glucose sensing. Such real-time monitoring tools represent a powerful diagnostic and monitoring tool for measuring glucose in diabetes research and point-of-care diagnostics [47].  Figure 3 shows the evolvement of the glucose biosensors from the first generation to the nanoglucose sensors, as explained by Chas and Clark [39].

Glucose biosensing with graphene has produced promising results as reported by Kang et al. [48]. In their work, they developed a graphene-based glucose biosensor using graphene and chitosan and drop-coated the mixture onto a glassy carbon electrode. Their graphene biosensor was able to measure glucose with a detection limit of 0.3603 mg/dl and a linear sensing range of 1.4412 mg/dl to 216.1871 mg/dl [48]. Likewise, Alwarappan et al. developed a polypyrrole (Ppy)-graphene biosensor by using the Ppy to encapsulate and trap graphene and glucose oxidase (GOx) on to a glassy carbon electrode [49]. Their biosensor was able to detect glucose with a limit of detection of 5.4 × 10^−2^ mg/dl and with a linear detection range of 3.60 × 10^−2^ − 0.7206 mg/dl [49]. Also, graphene fabricated with metal nanoparticles has been used to develop biosensors for glucose with very promising results, as reported by Lu et al. [50]. In their work, they used exfoliated graphite nanoplates (xGnPs) which they dispersed in ethylene glycol with a platinum (Pt) precursor, sonicated, and centrifuged to form xGnPs decorated with Pt nanoparticles. They then used Nafion to stabilize the nanoplates together with glucose oxidase. Their biosensors showed high glucose sensitivity with a detection limit of 1.80 × 10^−2^ mg/dl and a linear sensing range of 18.0156–360.3118 mg/dl. Another graphene-based glucose sensor was developed using chemical vapor deposition by Claussen et al. [51]. In their work, they grew multilayered graphene petal nanosheets (MGPNs) on a silicon-based surface for use in glucose biosensing. They used electrochemical deposition to deposit Pt nanoparticles on a 3D graphene petal followed by electrochemical deposition of the conductive poly(3,4-ethylenedioxythiophene)-poly(styrenesulfonate) doped with GOx. They then altered the size, density, and morphology of the Pt nanoparticles by changing the magnitude of the current pulse used to deposit the nanoparticles. Their biosensor was found to have a lower glucose detection limit (5.40 × 10^−3^ mg/dl) and wider linear sensing range (0.1801–900.7795 mg/dl) than nanostructured biosensors [51]. They then concluded that such a broad linear glucose sensing range could enable glucose monitoring in blood, saliva, tears, and urine, permitting new noninvasive sensing protocols for simultaneous glucose monitoring from numerous serum samples [51, 52].

Metal nanoparticles as mentioned before have been used to advance sensor performance and have therefore been used in the development of biosensors based on innovative detection principles, and as a result, they have also been used to improve glucose biosensors. For example, solution suspensions of nanoparticles have also been used to detect glucose via electrochemical and optical methods [47]. Researchers such as Rossi et al. [53] have capitalized on the ability of magnetic nanoparticles to be easily delivered and recovered in biomedical applications to immobilized enzymes for biosensor development. For example, in one of their researches, Rossi et al. immobilized glucose oxidase on 20 nm magnetite (Fe_3_O_4_) nanoparticles that were capable of detecting glucose concentrations up to 360.3118 mg/dl for 3 months when stored at 4°C [53]. Likewise, quantum dots were composed of manganese-doped zinc sulfide (ZnS), functionalized with glucose oxidase, and able to detect glucose at a detection limit of 0.0540 mg/dl and 2 linear ranges from 0.1802 to 1.8016 mg/dl and from 1.8016 to 18.0156 mg/dl via a phosphorescent detection mode [54].

Electrode surfaces have been used as sensor surfaces where metal nanoparticles can be immobilized and as such, wide variety of glucose biosensors with metallic nanoparticles and quantum dots have been developed [55, 57]. An array of nanoelectrodes created on a single electrode surface by separating nanoparticles or nanowires between nonconductive insulating materials such as alumina in an ordered manner have also been used for glucose biosensors due to their improved signal-to-noise ratio, enhanced mass transfer, and improved detection limits [58]. For example, an array of 250 nm in diameter platinum nanowires grown in the polycarbonate membrane via an electrodeposition method was developed by Yang et al. and was able to detect glucose with a wide linear range of between 0.018 and 540.477 mg/dl when functionalized with glucose oxidase [59] and was able to detect glucose in blood samples. Also, Wen et al. [60] developed a platinum carbon nanotube biosensor for glucose by inserting platinum nanoparticles into arrays of carbon nanotubes and functionalizing them with glucose oxidase. Their biosensor was found to be capable of detecting glucose with a detection range of between 0.0288 and 207.1793 mg/dl and with a low detection range of 0.9909 mg/dl [60]. In addition, more studies have revealed how nanoparticles electrodeposited on arrays of horizontally aligned carbon nanotubes grown from a porous anodic alumina template can be used to develop glucose biosensors [61, 62]. Claussen et al. electrodeposited platinum nanoparticles carbon nanotube arrays with distinct current densities which changed the density of the nanoparticles with glucose oxidase covalently linked to the particles to create enzymatic glucose biosensors [62]. The increased relative density of the platinum nanoparticles deposited on the carbon nanotubes improved the linear glucose sensing range from 5.4047–270.2334 mg/dl range to a 1.8016–360.3118 mg/dl range [62] as well as the detection limit which showed an improvement from 1.3331 mg/dl to 0.1045 mg/dl for their glucose biosensor [62]. In another study by Claussen et al., gold nanoelectrode arrays were able to detect glucose with a linear detection range up to 378.3274 mg/dl and detection limit of 1.8016 mg/dl [63]. In this study, gold nanowires were grown in the porous anodic alumina template via an electrodeposition method and covalently immobilizing glucose oxidase [63].

From what has been described above, nanotechnology has been able to improve the sensitivity and linear ranges of various glucose biosensors which is very important in point-of-care diagnostic devices.

### 2.3. Nanosensors for Point-of-Care Diagnosis of Infectious Diseases

Infectious diseases such as malaria, viral hepatitis, dengue fever, cholera, severe respiratory syndrome, and avian influenza are generally triggered by pathogenic microorganisms such as viruses, fungi, bacteria, and parasites that have a deep impact on humankind due to their distinctive characteristics such as their fast multiplication and unpredictability which sets them apart from the other diseases [64–67]. These diseases are a leading cause of death in developing countries [67, 68] where over 95% of these deaths are due to lack of proper diagnosis and treatment, such as difficulty in accessing adequate health care infrastructure [64, 69]. More importantly, the widespread infectious diseases have caused continuous increase of morbidity and mortality rates in the developing nations and can easily spread worldwide because of increased global travel [64, 70]. There is thus an urgent need to develop new and novel diagnostics tool for the detection of infectious diseases to stop the spread, secure public health, and promote treatment [64]. According to the World Health Organization (WHO), the ideal diagnostic device for infectious diseases should have high sensitivity, specificity, accuracy, robustness, user friendly, and inexpensive [64, 67, 71]. The conventional diagnosis techniques for these diseases include culture and microscopy, immunology, and the polymerase chain reaction (PCR) strategies [72–75]. Though these techniques have significantly contributed to the detection and diagnosis of infectious diseases and greatly promoted the prevention and treatment for various infectious diseases, they have shown several limitations such as inaccuracy and slowness, and they are expensive and require skilled expertise especially in developing countries [64]. This calls for the development of new and improved diagnostic techniques for early detection and high sensitivity and the potential for point-of-care tests (POCTs) to enable prevention and treatment of these infectious diseases among families and in community clinics worldwide [64, 76].

Due to the unique properties of nanomaterials in optical, mechanical, magnetic, catalytic, and electrical perspectives [64], advancement in nanotechnology has seen many applications especially in biomedical applications such as tissue engineering, drug delivery, bioimaging, and nanodiagnostics [77–79]. Because of their unique characteristics in early detection, high sensitivity, and potential for point-of-care tests, nanodiagnostics have attracted most attention for the diagnosis of infectious diseases [76] due to their potential to offer portability, robustness, and affordability. In this review, we focus on various nanodiagnostics devices which have been developed for point-of-care diagnostic of various infectious diseases.

In a study reported by Zhang et al. [80], they developed a simple and effective method which improved the detection sensitivity of dot-blot immunoassay by amplifying the reporting fluorescent signals with QD-nanobeads (QDNBs). They used the prepared QDNBs as amplified signal indicators and found that as low as 78 pg hepatitis B surface antigen (HBsAg) proteins could be detected in a one-step test [80]. Their results were also readable under a standard UV lamp illustration conditions obviating the need for complicated instrumentation and thereby providing the possibility for the development of QDNB-based POCT devices for hepatitis B [80]. Gold and silver metallic nanoparticles have also been used in the development of nanodiagnostics because they can emit intense absorption upon interaction with electromagnetic radiation [81]. Gold nanoparticles in solution change color from red to blue, and this has been used for nanodiagnostics since many different molecules, such as antibodies, antigens, and enzymes, can be conjugated with these gold nanoparticles as electrochemical labels, optical probes, and signal transfer amplifiers for the diagnosis of various diseases [64]. For example Darbha et al. [82] demonstrated the use of gold nanorods to diagnose HIV through their second-order nonlinear optical properties [82]. The gold nanorods demonstrated a rapid, simple, and efficient detection of single-base-mismatch HIV-1 virus DNA through the hyper-Rayleigh scattering (HRS) spectroscopy intensity changes [82]. Similarly, the HRS technique with gold nanoparticles was also developed to detect hepatitis C virus (HVC) infectious diseases in a study by Griffin et al. [83]. In their work, they conjugated gold nanoparticles with HCV ssRNA tagged with rhodamine 6G, through which as low as 80 picomolar HCV ssRNA was detected, and the selectivity was found to reach a single base-pair mismatch [82].

**Figure 4:**
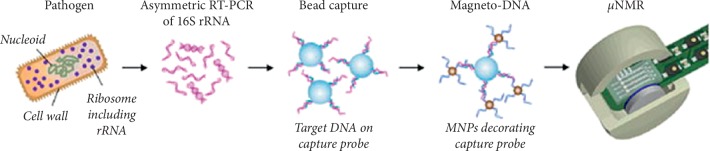
A schematic representation of the assay procedure where total RNA was extracted from the specimen and the bacterial 16S rRNA was amplified by asymmetric real time-PCR. Single-strand DNA of the amplified product was then captured by beads conjugated to capture probes, before hybridizing with magnetic nanoparticles (MNPs) to form a magnetic sandwich complex. Samples were subsequently analyzed using a *μ*NMR system. Reproduced from Chung et al. [84] with a Copyright Clearance Center's RightsLink® service and order number 4658140169987.

In another study by Chung et al. [84], they designed a dual probe-nanoparticle system capable of detecting and phenotyping common human pathogens where they prepared a nanoparticle assay which was based on a sandwich hybridization technique involving two specific oligonucleotide probes targeting the bacterial 16S rRNAs and designed to detect amplified target DNAs using a miniaturized nuclear magnetic resonance (NMR) device, as illustrated in Figure 4. They formed a magneto-DNA platform which allowed both universal and specific detection of various clinically relevant bacterial species, with sensitivity down to single bacteria [84]. The assay was found to be robust and rapid and simultaneously diagnosed with a panel of 13 bacterial species in clinical specimens within 2 hours [84] forming a generic platform which could be used to rapidly identify and phenotype pathogens for a variety of applications [84].

In yet another study by Lee et al., a handheld diagnostic magnetic resonance (DMR) system was developed for multiplexed, quantitative, and rapid analysis [85]. They used magnetic nanoparticles as a proximity sensor to magnify molecular interactions and found that the handheld DMR system could perform measurements on unprocessed biological samples [85]. They also demonstrated the use of the system for the detection and characterization of infectious agents, such as bacteria, viruses, and fungi, on a molecular level in real time, and measure a series of protein biomarkers in parallel [85]. They predicted that the predictable handheld miniaturized DMR platform, in combination with microfabrication strategies, could be used as a portable, low-cost, and high-throughput POC nanodiagnostics system for the large-scale detection of infectious diseases in the future [85].

Magnetic nanoprobes have also been used to develop a magnetic barcoding strategy for the detection of *Mycobacterium tuberculosis* (MTB) as reported by Liong et al. [86]. In their work, they developed a platform for the detection of nucleic acids based on a magnetic barcoding strategy where PCR-amplified mycobacterial genes were sequence-specifically captured on microspheres, labeled by magnetic nanoprobes, and detected by nuclear magnetic resonance [86]. They integrated all the components into a single, small fluidic cartridge for streamlined on-chip operation and used to detect MTB and identified drug-resistance strains from mechanically processed sputum samples within two and half hours [86]. The specificity of the assay was confirmed by clinically relevant non-MTB bacteria, and the clinical utility was demonstrated by the measurements from MTB-positive patient specimens. From their results, they concluded that if the magnetic barcode assay system can be combined with portable systems, then it has a potential of becoming a sensitive, high-throughput, and low-cost platform for point-of-care diagnostics for infectious diseases [86]. Similarly, this magnetic barcode system was also used to detect most representative infectious *Staphylococcus aureus*, methicillin-resistant *Staphylococcus aureus*, and *Klebsiella pneumoniae* bacteria as reported by Cihalova et al. [87]. In their work, they used fluorescent nanoparticle quantum dots (QDs) and magnetic particles to modify specific targeting bacteria-specific genes such as wcaG, fnbA, and mecA. From their results, they found that that platform had the ability to detect the infectious bacteria concentrations as low as 10^2^ CFU/mL [87], indicating that the portable magnetic barcode assay systems had potential in point-of-care diagnosis for the sensitive, efficient, rapid, and low-cost detection of many other infectious diseases [87].

### 2.4. Nanosensors for Point-of-Care Diagnosis of Malaria

Nearly half of world's population lives in malaria-endemic regions, and more than half a million deaths resulting from malaria and its complications are reported each year [88], making it a significant global health problem [89]. Significant achievements have been realized in malarial therapeutic development. However, eradication of malarial infection especially in low income areas has not achieved much success due to lack of early-stage diagnostic tools. Diagnosis of malaria involves identification and quantification of target metabolites (biomarkers) in biological fluids, mainly blood, urine, and saliva [88, 90]. A variety of biomarkers for malaria exist. Some of these include hemozoin which is a paramagnetic nanoparticle byproduct of the malaria parasite, also known as malaria pigment or malaria biomarker [90] and whose presence in the blood is indicative of malarial infection, plasmodium falciparum histidine-rich protein 2 (PfHRP 2) [91, 92], and topoisomerase I expressed by the malaria causing *Plasmodium* parasite [88]. The use of hemozoin is highly recommended as a biomarker in the development of malarial diagnostic devices because it is more stable, cheaper, and easily available compared to PfHRP 2 [90]. It has further been established that hemozoin is chemically and structurally similar to *β*-hematin [90]. For this reason, most authors use *β*-hematin to mimic hemozoin in the development of malarial sensor devices.

An ideal point-of-care diagnostic device should detect at least 100 parasites/*μ*L of blood, which is the threshold for early-stage malaria infection [89]. This area is therefore attracting a lot of research interest, and a few researchers have proposed potential tools for point-of-care malarial diagnostics. In one of the reports, a portable optical diagnostic device for malaria was designed by Armani et al. [89] using *β*-hematin. Using the device, the authors demonstrated its potential use in early point-of-care diagnosis of malaria by detecting *β*-hematin in whole rabbit blood. The limit of detection achieved was less than 8.1 ng/mL in 500 *μ*L of blood, corresponding to less than 26 parasites/*μ*L.

A recent study by Obisesan et al. [90] demonstrated the use of gold electrodes modified with metal nanoparticles, to develop electrochemical sensor devices for the detection of *β*-hematin in blood samples from mice. The nanoparticles used were specifically CuO, Al_2_O_3_, and Fe_2_O_3_, each synthesized using chemical and microwave methods. Each of the nanoparticle-modified electrode surfaces acted as platforms on which electrocatalytic reduction of *β*-hematin in the blood sample occurred. The chemically synthesized CuO nanoparticles yielded higher electrocatalytic currents than the microwave-synthesized CuO nanoparticles. Both Al_2_O_3_ and Fe_2_O_3_ produced lower catalytic currents compared to the CuO nanoparticles but at lower potentials. The authors found a more favourable electrocatalytic reduction of *β*-hematin on CuO-modified gold electrodes, both chemically synthesized and microwave synthesized. Furthermore, the CuO-modified gold electrode exhibited high stability and good selectivity to the *β*-hematin compared to *S. typhi* antiserum VI typhoid biomarkers. In fact, the sensor afforded simultaneous detection of *β*-hematin and *S. typhi* antiserum VI with well-defined peaks, having a peak separation of 250 mV in serum. The limits of detection (LOD) and limits of quantitation (LOQ) obtained by these authors are summarized in Table 1.

**Figure 5:**
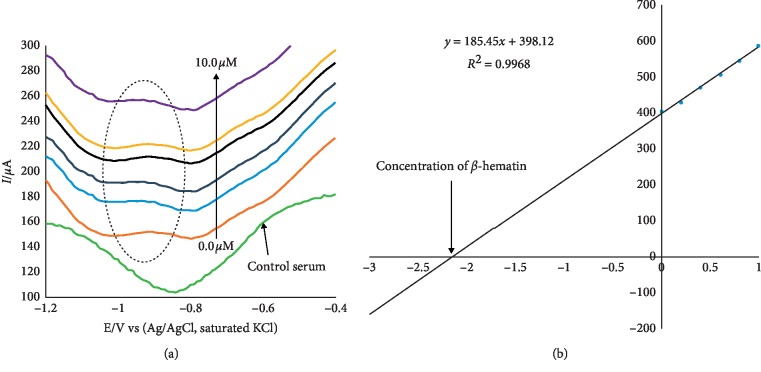
(a) Typical square wave voltammetry (SWV) analysis of *β*-hematin in the mice serum sample; (b) calibration curve for *β*-hematin determination in the unspiked human urine sample using the standard addition method. Reproduced from Obisesan et al. [90], an open-access article distributed under the terms of the Creative Commons Attribution License (CC BY) which permits the use, distribution, or reproduction in other forums provided the original author(s) and the copyright owner(s) are credited and that the original publication in this journal is cited, in accordance with accepted academic practice.

In a further study, the Au-CuO modified electrode was used for detection of *β*-hematin in the serum of infected mice and human sera diagnosed with malaria parasite using the square wave voltammetry technique (SWV). The procedure followed the standard addition method, and the results indicated that the *β*-hematin peak was observed at around −0.80 V in animal serum and −0.91 V in human serum. This peak was absent in animal serum that was not infected with malaria parasite shown as control serum, in Figure 5(a). After spiking the infected serum samples with standard concentrations of *β*-hematin, their results indicated a current response increase with increasing *β*-hematin concentration and the current plot vs. concentration was obtained, as shown in Figure 5(b). These results indicated percentage recoveries within the accepted recovery range (75–110%) for a reliable analytical device. Thus authors demonstrated that the sensor would quantitatively detect malaria parasites in human serum within allowable limits.

Despite their great potential in fabrication of point-of-care diagnostic devices, malaria nanosensors have not been greatly explored. The discussion above indicates only a few publications (two recent) on the use of nanosensors for the detection of malaria, whose potential has been clearly demonstrated. A lot of research is therefore needed to improve the demonstrated nanosensors and upscale their applicability away from the laboratories to the hospitals.

### 2.5. Nanosensors for Point-of-Care Diagnosis of Human Immunodeficiency Virus (HIV)

Human immunodeficiency virus (HIV) is a major worldwide public health issue which calls for refined clinical management [93]. It has developed to a multisystem condition involving the cardiovascular disorders (CVDs) and rheumatoid arthritis (RA) [94]. These cardiovascular disorders (CVDs) in HIV patients are the number one cause of morbidity and mortality [94]. In most cases, HIV is detected by monitoring the viral load [95] and early detection of HIV infection is the best way to prevent its spread and to improve the efficiency of the antiretroviral therapy [96]. The traditional methods for the detection of the HIV viral load include culturing, enzyme-linked immunosorbent assay (ELISA), and polymerase chain reaction (PCR). Nucleic acid amplification tests (NAATs) are usually used as the gold-standard method for detecting low concentrations of the virus in blood [96]. Unfortunately, these methods face various challenges when it comes to point-of-care implementation [95, 96] since some of them are time-consuming, costly, and technically require skilled technicians. Though there have been remarkable efforts to develop new strategies for detection and treatment of HIV, translating these strategies into resource-limited settings has been found to be challenging [93]. Several researchers have therefore devoted their efforts to develop point-of-care diagnostic devices to monitor the HIV viral load with high sensitivity by leveraging micro- and nanoscale technologies with the aim of applying them to monitor antiretroviral therapy and early infant detection of HIV. In this review, we focus on these new strategies put in place to develop nanosensors for point-of-care diagnosis of HIV viral load.

**Figure 6:**
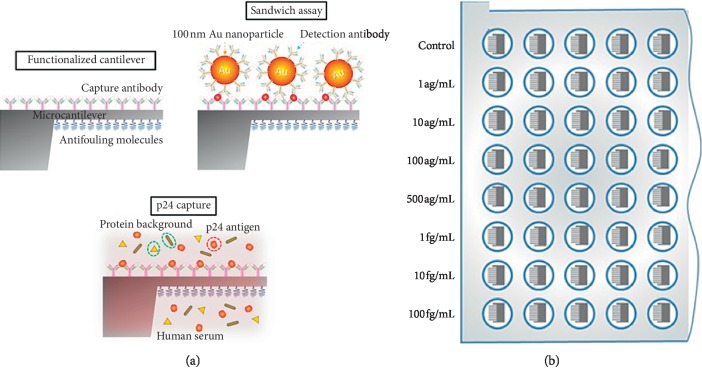
A schematic representation of the p24 sandwich immunoassay: (a) the top surface of the cantilever is functionalized with capture antibodies against HIV-1 p24 antigen. Antifouling molecules are immobilized on the bottom surface of the cantilever and voids between the antibodies to minimize nonspecific interactions. The cantilever is then immersed in the human serum sample to allow specific binding of p24 to the cantilever surface (middle schematic). Finally, the p24 antigen captured on the cantilever is specifically linked to 100 nm-diameter gold nanoparticles that carry detection antibodies. (b) Schematics of the 96-well microtiter plate format, in which the immunoassays were carried out. Reproduced from Kosaka et al. [96] an open access article distributed under the terms of the Creative Commons Attribution License, which permits unrestricted use, distribution, and reproduction in any medium, provided the original author and source are credited.

A nanoplasmonic-based sensor for the detection of HIV at clinically relevant concentrations has been reported by Inci et al. [95]. In their work, they developed a sensing platform which was based on the unique nanoplasmonic properties of nanoparticles by utilizing immobilized antibodies to selectively capture rapidly evolving viral subtypes. The nanoplasmonic platform then measured shift in signal which is caused by the viruses captured on a gold nanoparticle coated surface. They used spiked whole blood samples and clinical discarded HIV-infected patient whole blood samples validated by the RT-qPCR to demonstrate the capture, detection, and quantification of various HIV subtypes (A, B, C, D, E, G, and subtype panel). Their results showed high repeatability, sensitivity, and specificity down to 98 ± 39 copies/mL (*i.e*., subtype D). The assay time was within 1 hour for capture and 10 minutes for detection and data analysis. Their results also indicated that detection of viruses from unprocessed whole blood samples directly from patients was feasible, and their platform technology could enable rapid isolation, capture, detection, and quantification of viruses, thus allowing for direct multiple pathogen detection which can be termed as a significant step towards providing POC tests at resource-constrained settings as well as at the hospital and primary care settings [95]. Also, in another research by Kosaka et al. [96], they reported how they developed a sandwich immunoassay by combining nanomechanical properties of gold nanoparticles and optoplasmonic transduction methods for the detection of the HIV-1 capsid antigen p24 in human serum, as shown in Figure 6. In their work, the gold nanoparticles were used as both mechanical and plasmonic labels, while a compliant microcantilever was used as both a mechanical resonator and an optical cavity for the transduction of the mechanical and plasmonic signals. Their results for the immunoassay indicated a limit of detection of 10−17 g/mL that was equivalent to one virion in 10 mL of plasma translating to 5 orders of magnitude better than last generation of approved immunoassays and 2 orders of magnitude better than NAAT. From their results, they concluded that their technology met the demands to be produced en masse at low cost and the capability for miniaturization to be used at the point-of-care [96].

Graphene has also been used to develop nanosensors for the detection of HIV, as reported by Islam et al. [94]. In their study, they developed a graphene nano-based electrochemical sensor for detection of HIV and related diseases such as cardiovascular disorders and renal arthritis. They functionalized graphene with amines and covalently conjugated them with various antibodies such as anti-p24 for HIV, anti-cardiac troponin 1 (anti-cTn1) for CVDs, and anti-cyclic citrullinated peptide (anti-CCP) for RA via carbodiimide activation to detect various biomarkers. They then characterized the graphene-antibody conjugate using various techniques such as UV-Vis, Raman spectroscopy, scanning electron microscopy, and atomic force microscopy. The interaction of the biomarkers with the conjugated antibodies was evaluated for its electrochemical performance with respect to resistance and electrode surface changes. Their results indicated high sensitivity with a good linear response to p24, cTn1, and CCP from 1 fg/mL to 1 *μ*g/mL with a limit of detection (LOD) of 100 fg/mL for p24 and 10 fg/mL for cTn1 and CCP under standard optimized conditions. They thus concluded that the graphene-nano-based sensor demonstrated excellent performance to be used for the on-site detection of HIV, CVD, and RA biomarkers in real samples [94].

In yet another study by Ng et al. [97], they demonstrated the use of a point-of-care system that utilized magneto-nanosensor arrays and magnetic nanoparticles for the detection of HIV in saliva and leukocytosis in plasma and whole blood. In their work, they used diagnostic chips which consisted of 80 individual analytes in a single sample which could potentially detect lower levels of antigen enabling early detection of HIV in a noninvasive manner [97]. Their magneto-nanosensor was mobile-based diagnostic platform complete with the circuitry, signal processing, user interface, and mobile application for the point-of-care usage settings with low standard deviations and quantification of analyte with reduced electrical noise. As a proof of concept, their nanodevice was able to quantitatively detect HIV in saliva and leukocytes in plasma at a point of care within 16 minutes of assay time with an accuracy of 90% and 80%, respectively [97]. They then concluded that the portability, high sensitivity, and ease of use of their nanodevice has the potential to be used for point-of-care diagnosis of HIV and hence enable early detection of the diseases.

### 2.6. Nanosensors for Point-of-Care Diagnosis of Bilharzia

Bilharzia, also known as schistosomiasis, is a very debilitating disease which affects more than 200 million people and whose highest burden of morbidity and mortality is found in African countries [98]. Even though it has an enormous effect on the health and socioeconomic burden of the society, it still remains a neglected tropical disease, with limited attention from governments and stakeholders in health care [98]. In addition to these negative direct impact on health, it also fuels the vicious circle of poverty and stigma that leaves people unable to work, go to school, or participate in family and community life [99] and it is caused by the schistosome parasite [100, 101]. One of the critical areas which is hugely disadvantaged is the development of accurate and sensitive diagnostics tools [98].

Diagnosis of schistosomiasis is imperative for the detection and treatment of the disease in endemic and nonendemic settings since case detection, assessment of morbidity, and the evaluation of control strategies are all build on the results from diagnostic results [98, 102]. Current diagnostic methods for bilharzia are use of the microscopic determination of parasite eggs (in urine or stool) or by immunological methods (antibody or antigen detection) [102–105]. In addition, the sensitivity of the examinations also depends on the severity of the infection. For example, in low-grade infections, the sensitivity of one microscopic examination may be as low as 20%, and in clinically suspected cases, up to 5 urine specimens (collected over midday) and or 5 stool specimens for microscopic examinations are recommended to increase the sensitivity of the tests [102, 106]. Also, depending on the methodology used and the timing in the postinfected host, the sensitivity of current antibody assays is not optimal (ranging from 65 to 86%) [102, 106]. Some of the commonly used methodologies are based on detection of antibodies directed against the soluble egg antigen (SEA) and due to the retention of the eggs and constant secretion of the SEA by the deposited eggs; antibodies may be elicited for an indefinite period after the primary infection, irrespective of successful treatment. However, these methods are not very sensitive and are unreliable [107, 108]. A conclusive detection method is therefore an indispensable part of treatment, both in the clinic and during mass drug administration (MDA), for the monitoring efficacy of treatment [98].

Nanotechnology has been used to improve the sensitivity since it has the potential to offer not only improvement to current approaches but also unexpectedly delivers many new tools and capabilities [109–111]. The application of nanoparticles in immunosensing has shown great potential in developing versatile point of care diagnostic devices which are highly sensitive [112–115]. In this part, we will explore various nanosensors which have been developed for the detection of bilharzia.

**Figure 7:**
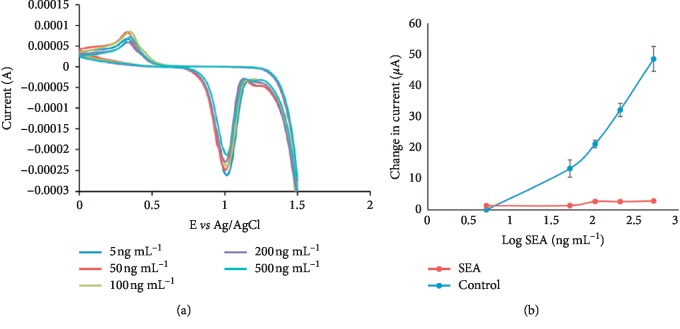
(a) Representative cyclic voltammograms obtained after incubating the AuNPs-rabbit anti-bilharzia antibody modified screen-printed gold electrodes (Model DS 250BT) in varying concentrations of SEA, *n* = 3. (b) SPE (Model DS 250BT) nano-biosensor CV calibration curve for the change in anodic peak currents *versus* log of concentration of SEA, *n* = 3. Reproduced from Odundo et al. [102], an open access article under the Creative Commons Attribution (CC BY) license which permits free immediate access to, and unrestricted reuse of, original works of all types as long as the author and original source are properly cited.

A study by Odundo et al. [102] describes the use nanotechnology to develop a simple and highly sensitive nanostrip, consisting of gold nanoparticles conjugated with bilharzia antibody and demonstrated its potential for diagnosis of soluble egg antigen (SEA) a bilharzia antigen. They used cyclic voltammetry to characterize their nanosensor, and their results are shown in Figure 7, which indicated an increase of current with the increase in the SEA concentration. Their results also indicated a limit of detection of 8.3887 × 10^−2^ ng/ml which was 80% better than that obtained when using the gold and glassy carbon electrodes. Also, the developed nanostrips were able to detect the bilharzia antigen in 30 stool samples collected from a bilharzia endemic area in Kenya, and their results indicated a positive response of between 1.13 × 10^1^ ng/ml to 2.3 × 10^3^ ng/ml of bilharzia antigen. From their results, they concluded that the strips can detect bilharzia antigen in real samples and can therefore be used for point of care devices for the detection of bilharzia [102]. In another study by Shohayeb [116], they report the development of a novel screen-printed immunosensor for detection of Schistosoma mansoni antibodies (ABs). In their work, they fixed soluble worm antigens (SWAs) onto the nanocarbon working area of a screen-printed electrode using glutaraldehyde-chitosan cross-linkers and then evaluated the binding of the Schistosoma mansoni antibody to the antigen-loaded screen-printed electrode by cyclic and differential pulse voltammetry. Their results gave a calibration curve for Schistosoma mansoni ABs binding to the SWA-loaded screen-printed electrode with a reproducible linear range at a concentration ranging between 0.038 and 20 ng/ml. This quantitative response obtained at nano-level amounts of the AB made them to conclude that their method could be used in the future to develop a disposable screen-printed electrode for diagnosis of schistosome infections [116].

Kamel et al. [117] in yet another study demonstrated the use of gold nanoparticles to improve the sensitivity and specificity of a sandwich enzyme linked immunosorbent assay (ELISA) in the detection of human schistosoma. In their work, they conjugated gold nanoparticles with anti-schistosomal monoclonal antibody (MAb) and evaluated the sensitivity and specificity in diagnosing human Schistosoma mansoni infection [117]. They used serum samples of 116 subjects which included 71 mansoni infected patients, 25 patients infected with parasites other than schistosomiasis, and 20 uninfected healthy individuals. They further subdivided the patients infected with mansoni according to egg count in their stool samples into light, moderate, and severe infection. They then compared their results to those after using the MAb sandwich ELISA system. Their results indicated that the AuNPs-MAb/ELISA reached a lower detection limit of 10 ng/ml compared to 85 ng/ml on using MAb/ELISA, and the optimal concentration of AuNPs-MAb used was 12-folds less than that of Mab [117]. The gold nanoparticles were found to improve the sensitivity and specificity of the ELISA for detecting circulating schistosomal antigen (CSA) by 100% and 97.8% as compared to 87.3% and 93.38%, respectively, when the ELISA was done without the gold nanoparticles. They then concluded that conjugating the gold nanoparticles with MAb increased the sensitivity and specificity of sandwich ELISA for detection of CSA and thus active and light infections could be easily detected [117]. They further concluded that the binding could also decrease the amount of MAb consumed in the assay and hence lower the cost [117]. From the discussion above, it is very clear that nanotechnology enhances the sensitivity and specificity of the existing sensors for the detection of bilharzia which can be very important in the development of point-of care devices for bilharzia.

## 3. Discussion

Significant achievements have been realized in the development of nanosensors for point-of-care diagnostics for cancer, diabetes, malaria, HIV, and bilharzia. At least for each of these diseases and medical conditions, a nano-based sensor/biosensor with desirable clinical characteristics has been reported. Several authors have demonstrated potential applications of the developed nanosensors using real and/or simulated samples and the results point towards great success. For example, nanotechnology has been found to improve the sensitivity and linear ranges of various glucose biosensors which is very important in point-of-care diagnostic devices.

However, some diseases have received little attention in the application of nanotechnology for their diagnostics and detection. For instance, there is very little application of nanotechnology in the development of sensors for point-of-care cancer diagnostics. In this review, only ovarian cancer, breast cancer, and angiosarcoma (blood and lymph vessels cancer) are reported to have had nanosensors developed for their detection. The attention given to ovarian cancer and breast cancer presents a milestone in containing the two types of cancers, which are life-threatening in women. There is need to develop nanosensor platforms for the other prostate cancer, esophageal cancer, colorectal cancer, lung cancer, lymphoma, skin, and liver cancer, which are causing significant deaths, as opposed to angiosarcoma, which is a rare type of cancer. Also, only two publications have clearly demonstrated the potential of nano-based biosensors for the detection of malaria. The nanosensor platforms so far reported are limited to only a few types of nanomaterials. It would be greatly desirable to explore other types of nanomaterials, with superior properties to improve sensor performance. Despite the great potential of nanotechnology in the field of disease diagnostics, none of the demonstrated devices discussed in this review have been improved into prototypes in preparation for commercialization.

Another challenge facing the devices discussed here is the sample used in diagnosis. The diseases and medical conditions discussed here mainly use blood as the diagnostic sample, which presents a painful sampling process. Moreover, the patient has to be awake for the sample ton be collected. Some authors have demonstrated that saliva, tears, and urine can potentially replace blood samples in the diagnosis of malaria and detection of blood sugar levels. However, no further research on these devices has been made to explore possibilities of commercializing them. Future research should therefore focus on developing and commercializing more portable hand-held glucose sensors that uses either saliva, tears, or urine, which are not painful to obtain from patients. Further research to introduce wireless noncontact clinical glucose biosensors will greatly enhance glucose monitoring to patients, even when asleep.

## 4. Conclusion

In order to provide better quality health care, high standard of health care management have to be achieved, and thus development of nanosensors for point-of-care diagnostics is an important area of research. Nanotechnology has been able to improve the sensitivity and linear ranges of various diseases as discussed in the review which is very important in point-of-care diagnostic devices. Though progress in this field is gaining momentum and several researchers have devoted their time to develop novel nanosensors for point-of-care diagnosis of various diseases, the ultimate goal of achieving long-term, accurate, and continuous monitoring in patients has not yet been reached. For example, our review only indicated that a few publications (two recent) on the use of nanosensors for the detection of malaria, whose potential has been clearly demonstrated. Moreover, the applicability of the fabricated sensor for onsite cancer diagnosis, cost, and accessibility to rural patients are aspects that require further research, improvements, and tested for potential use. A lot of research is therefore needed to improve the demonstrated nanosensors and upscale their applicability away from the laboratories to the hospitals.

## Figures and Tables

**Table 1:** Summary of limits of detection and limits of quantitation obtained on catalytic reduction of *β*-hematin on CuO, Fe_2_O_3_, and Al_2_O_3_ each synthesized using chemical and microwave methods.

CuO				Al_2_O_3_				Fe_2_O_3_			
Chemical synthesis		Microwave synthesis		Chemical synthesis		Microwave synthesis		Chemical synthesis		Microwave synthesis	
LOD	LOQ	LOD	LOQ	LOD	LOQ	LOD	LOQ	LOD	LOQ	LOD	LOQ
0.83	2.52	0.83	2.52	0.71	2.15	0.43	1.30	1.08	3.02	0.77	2.32

All values are in *μ*g/mL [90].
